# Morphometric, Biomechanical and Histologic Assessment of Physiologic Ovine Cervical Intervertebral Disc: An Experimental Study and Brief Literature Review

**DOI:** 10.3390/ijms252312579

**Published:** 2024-11-22

**Authors:** Nikolaos Gkantsinikoudis, Savvas Koltsakidis, Panagiotis Prodromou, Eleni Aggelidou, Stylianos Kapetanakis, Eleftherios Tsiridis, Ioannis Magras, Dimitra Psalla, George Kazakos, Dimitrios Tzetzis, Aristeidis Kritis

**Affiliations:** 1 Department of Physiology and Pharmacology, School of Medicine, Faculty of Health Sciences, Aristotle University of Thessaloniki (A.U.Th.), 54124 Thessaloniki, Greece; nikgkantsinikoudis@gmail.com (N.G.); angelide@auth.gr (E.A.); 2 Regenerative Medicine Center, Department of Basic and Translational Research of Special Unit of Biomedical Research and Education, School of Medicine, Faculty of Health Sciences, Aristotle University of Thessaloniki (A.U.Th.), 54124 Thessaloniki, Greece; 3 Digital Manufacturing and Materials Characterization Laboratory, School of Science and Technology, International Hellenic University, 57001 Thessaloniki, Greece; skoltsakidis@ihu.edu.gr (S.K.); prodromo1997@gmail.com (P.P.); d.tzetzis@ihu.edu.gr (D.T.); 4 Spine Department and Deformities, Interbalkan European Medical Center, 57001 Thessaloniki, Greece; stkapetanakis@yahoo.gr; 5 Academic Orthopedic Department, Papageorgiou General Hospital, Aristotle University School of Medicine, 56403 Thessaloniki, Greece; etsiridis@auth.gr; 6 Second Department of Neurosurgery, Hippokration General Hospital, Aristotle University School of Medicine, 54642 Thessaloniki, Greece; john.magras@gmail.com; 7 Laboratory of Pathology, Faculty of Veterinary Medicine, Aristotle University of Thessaloniki (A.U.Th.), 54124 Thessaloniki, Greece; dpsalla@vet.auth.gr; 8 Companion Animal Clinic, School of Veterinary Medicine, Aristotle University of Thessaloniki (A.U.Th.), 54627 Thessaloniki, Greece; gkdvm@vet.auth.gr

**Keywords:** sheep, ovine, intervertebral disc, cervical, anatomy, biomechanics

## Abstract

The ovine cervical spine model has been established as a representative model of the human cervical spine in the current literature, and is the most commonly used large animal model in studies investigating pathogenesis and treatment strategies for intervertebral disc (IVD) degeneration. However, existing data regarding morphometry, biomechanical profiles and the microscopic features of a physiological ovine cervical IVD remain scarce. Hence, the aim of this study was to perform a multimodal morphometric, biomechanical and histologic evaluation of a normal ovine cervical IVD. For this purpose, nine ovine cervical IVDs were harvested from three female sheep, and subjected to morphometrical, biomechanical and histologic analyses. The biomechanical assessment included the performance of cyclic compression, creepand compressive strength tests in a controlledlaboratory environment. Histological evaluation was performed using hematoxylin–eosin, Masson’s trichrome and Alcian blue staining. The results from the morphometric analysis showed that the range of disc heights was 4–9 mm in all surfaces, featuring a constant increase from cranial to caudal levels. Biomechanical evaluation revealed that cyclic loading for 20 cycles was necessary for preconditioning so that the repeatability of the force–displacement hysteresis response is present. The critical failure point was defined at 15.5 MPa, whereas Young’s modulus of elasticity was 1.2 MPa. The histologic assessment demonstrated the presence of a concentric arrangement of collagen lamellae in external annulus fibrosus, along with the sparsely organized internal nucleus pulposus. Ovine cervical IVD represents a complex structure with distinct features that should be considered by researchers in this field in order to optimize the reliability and validity of testing results.

## 1. Introduction

The intervertebral disc (IVD) represents a normal anatomic spine structure primarily responsible for the distribution of axial loads and the maintenance of physiological mobility [1]. From a clinical point of view, knowledge of the IVD characteristics constitutes a veritable necessity since the emergence of the clinically remarkable degeneration of IVD, widely known as degenerative disc disease (DDD), continues to represent the primordial etiology of chronic neck and lower back pain in current years [1,2,3,4]. Despite the ever-growing incidence of DDD and the associated major socioeconomic sequelae, currently utilized therapeutic modalities remain limited and incapable of interrupting the progressively deteriorating natural course of the underlying disease, highlighting the rather urgent need for the development of novel efficacious modalities [1,5].

Considering the aforementioned data and the requirement for the expansion of experimental IVD research for the subsequent development of clinically beneficial products, there has been much effort towards the determination of an ideal large animal model in IVD research, considering that homolog cadaveric human spine specimens are characterized by certain limitations such as the variability in epidemiologic and biologic core features, i.e., age, gender and grade of degeneration, and sourcing difficulties due to practical and ethical reasons [6]. Within this literature, the ovine cervical spine model has been established as an acceptable homolog for relative invitro studies, and is now the standard large animal model that is utilized [6,7,8,9,10,11]. Until now, ovine cervical IVD spine models have been extensively utilized in numerous studies evaluating implants for cervical disc replacement [12], cervical spine fusion [13,14,15], the investigation of the biologic effects of interventional methods [16,17,18], as well as tissue-engineering (TE) applications [19,20].

Despite the plethora of published reports regarding preclinical evaluation of spine implants in ovine cervical IVD models, there is a considerable paucity of literature data regarding the delineation of normal ovine cervical IVD morphometry, its biomechanical profile and histological characteristics [21]. Furthermore, the authors in the vast majority of these investigations assessed limited outcome measures and no studieshave attempted to provide a multimodal assessment of physiological ovine cervical IVDs at the morphometric, biomechanical and microscopic levels [22,23,24,25,26,27,28,29,30,31]. Hence, previous studies have primarily focused on isolated parameters related to IVD morphometry, biomechanical properties or histological features. Consequently, an integrated analysis of these three characteristics, aimingto correlate data and derive practical conclusions, has not been conducted yet.

The aim of the present study was to conduct a holistic morphometric, biomechanical and histologic assessment of normal ovine cervical IVD as a unified structure. The results of our study are analytically discussed within the pertinent literature framework in the context of a brief literature review. Particularly, specific emphasis is given topresenting data under a TE perspective, considering that meticulous knowledge regarding the baseline characteristics of utilized animal models represents a prerequisite prior to rational in vivo investigation for researchers in this field.

## 2. Results

### 2.1. Morphometric Assessment

All harvested specimens (*n* = 9) were considered eligible according to macroscopic examination, after being subjected to multimodal assessment as analyzed above. Morphometric measures were comparatively evaluated via statistical analysis across cervical spine units. The analysis demonstrated that anteroposterior (AP) and transverse (T) diameters in conjunction with measured disc heights in all sides featured a constant increase in a cranio–caudal direction. The results of the statistical analysis of recorded values are analytically presented in Table 1. Statistical analysis also demonstrated that retrieved values from the vast majority of measured parameters followed normal distribution (Table 1).

### 2.2. Biomechanical Assessment

**Figure 1:**
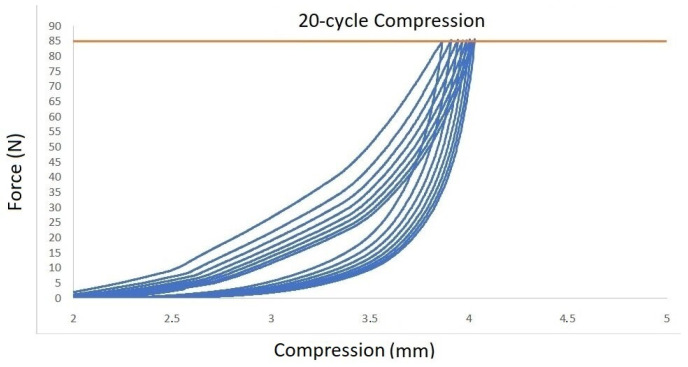
Cyclic compression testing the loading–unloading diagram.

**Figure 2:**
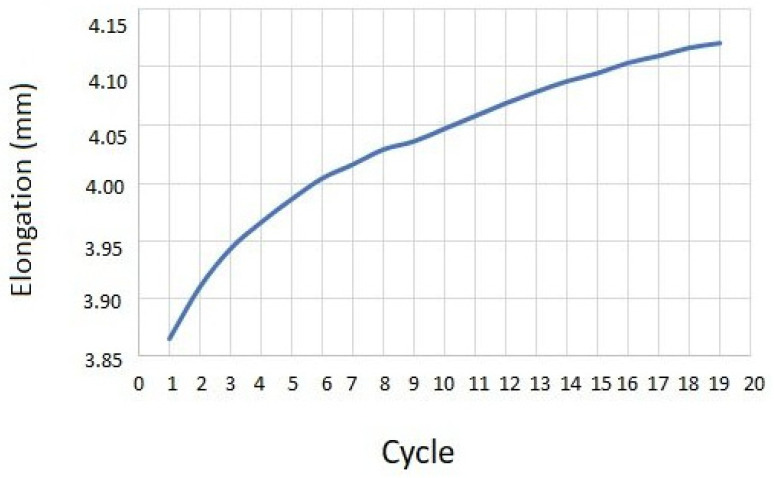
Schematic representation of recorded elongation per cycle in compression cyclic testing.

**Figure 3:**
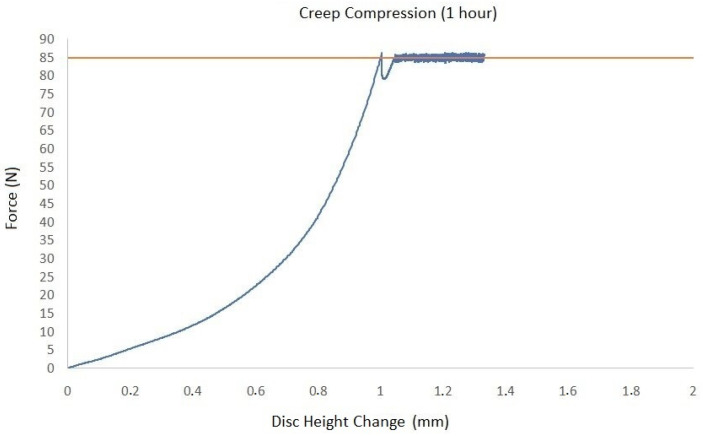
Schematic representation of creep testing results (brown horizontal line: peak of applied force).

Regarding biomechanical evaluation, the representative results for compression cyclic testing for a single specimen (C3–C4) are presented in Figure 1. It was demonstrated that cycle loading was necessary for preconditioning so that the repeatability of the force–displacement hysteresis response is ensured. In the first loading cycle, the disc was compressed by 3.85 mm. This value tended to progressively increase, reaching a plateau of 4.1 mm at the 20th cycle (Figure 2). Creep behavior was also investigated with results being demonstrated in Figure 3. In this testing procedure, the constant application of compressive force to a plateau of 85 N led to the gradual compression of 1.35 mm after 1 h (Figure 3).

**Figure 4:**
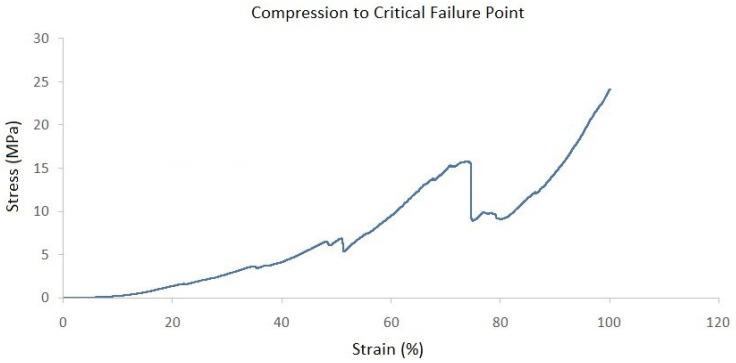
Recorded stress–strain curve after compression to critical failure testing.

Finally, the ultimate compression strength of evaluated specimens was analyzed by the application of a gradually increasing compressive load. The results demonstrated that the first non-critical failure occurred at 1.5 MPa, followed by several additional small peak drops. The critical failure was finally identified at 15.5 MPa, which was characterized by a sharp decrease in registered stress (Figure 4). Thereby, Young’s modulus of elasticity was calculated to be 1.2 MPa.

### 2.3. Histological Assessment

**Figure 5:**
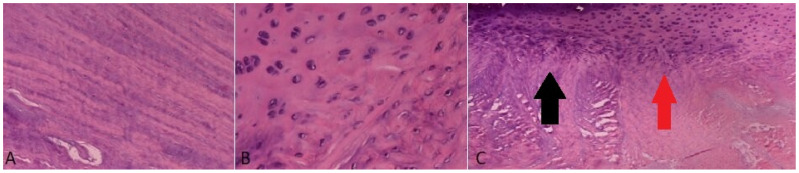
Hematoxylin–eosin staining of a standard prepared specimen. (**A**) Well-organized annulus fibrosus with concentric collagen lamellae (magnification: 40×); (**B**) Sparsely organized nucleus pulposus with notochordal cells and extracellular matrix (magnification: 100×); (**C**) Cartilaginous endplate–nucleus pulposus junction (black arrow), with bridging Sharpey fibers (red arrow) (magnification: 10×).

**Figure 6:**
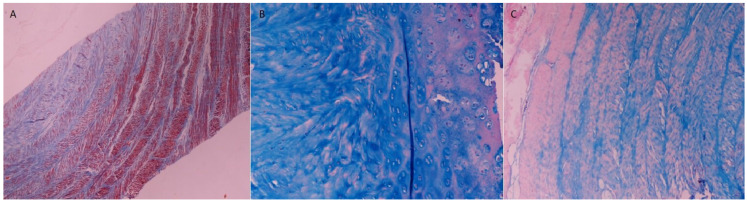
Masson’s trichrome (**A**) and Alcian blue (**B**,**C**) staining of a standard prepared specimen, demonstrating the concentric collagen lamellae ((**A**), magnification: 4×), the rich in proteoglycan extracellular matrix of nucleus pulposus ((**B**), magnification: 40×) and their progressive reduction along the periphery of the specimen ((**C**), magnification: 10×).

The microscopic evaluation of the specimens (*n* = 2) was conducted with hematoxylin–eosin (Figure 5), Masson’s trichrome and Alcian blue (Figure 6) staining in a conventional optical microscope. Hematoxylin–eosin staining demonstrated the presence of a well-organized concentric network of collagen fibers in the outer IVD portion in conjunction with the depiction of notochordal cells with a mild proliferation activity within a sparse extracellular matrix in central IVD portion representing annulus fibrosus and nucleus pulposus, respectively. Moreover, the microscopic examination revealed the presence of collagenous fibers that connected the nucleus pulposus to adjacent cartilaginous endplates, representing Sharpey fibers (Figure 5).

Masson’s trichrome staining highlighted the architecture of collagen lamellae in the annulus fibrosus, whereas Alcian blue staining displayed the semantic concentration of proteoglycans in the cartilaginous nucleus pulposus, with a gradually decreasing concentration from the central to peripheral portions of disc, reflecting the relative paucity of annulus fibrosus in these molecules (Figure 6).

## 3. Discussion

Sheep represent a dominantly selected large animal model in IVD research in recent years [9,21]. Reitmaier et al. conducted a systematic review in order to investigate the features of recruited animal models in IVD preclinical studies. After the analysis of 322 in vivo investigations, they found that ovine models represented the preponderant selection among different studies, being selected in 54% of cases. These studies primarily included fusion experiments followed by degeneration and surgical approaches studies. Nevertheless, it was stated that the selection of homolog models in these studies primarily relied on the researcher’s preferences and knowledge, given the relatively limited scientific value of existing literature reports [9].

Despite the proven wide recruitment of ovine cervical spine models for the investigation of various pathologic conditions and novel implants in the preclinical framework [8,15,18,20], published literature data regarding physiologic anatomy and the biomechanical behavior of the ovine cervical intervertebral disc remain scarce. Particular research groups attempted to shed light on this crucial issue by designing experimental investigations on the morphometric assessment of normal cervical ovine IVD, with or without the comparison with respective data obtained from human cadaveric studies [22,23,24]. The principal features of these studies are represented in Table 2.

Wilke et al. were the first to conduct a detailed morphometric evaluation of the physiologic ovine cervical spine. For this purpose, five total spine specimens harvested from female Merino sheep were evaluated. A plethora of diameters in vertebrae in conjunction with the anterior height of IVDs were analytically reported. Anterior disc height was depicted to be greatest in the cervical spine (between 6.8 ± 0.6 and 7.2 ± 0.8 mm) and least in the thoracic spine. Hence, the correlation of study findings with those of the published literature data advocated for the numerically greater disc thickness of ovine cervical IVDs in contrast to those of humans, a difference designated to be about 2–3 mm. Considering overall outcomes, the authors concluded that ovine spine models may represent a valid model for the preclinical in vivo testing of spinal implants [24].

In the last published investigation, Kandziora et al. endeavored to provide deeper insightsinto the physiologic ovine cervical spine. Therefore, they opted to conduct a comparative analysis of twenty fresh adult ovine cervical spine specimens versus twenty adult fresh human specimens. The measurement of the disc space height was calculated with a digital ruler in conventional radiographic views prior to dissection, whereas the authors attempted also to determine cervical spine lordosis, also conducting comparative bone mineral density measurements. Radiographic results demonstrated that the mean disc space height was depicted to increase in the cranio–caudal direction, whereas the respective posterior remain unaltered. Morphometric analysis demonstrated that anterior vertebral body height in ovine specimens was consistently greater than the respective posterior vertebral body height, which is associated with a disproportion between the lower and upper endplates, a characteristic not encountered in human specimens. Furthermore, ovine vertebrae demonstrated greater height in contrast to human ones [22].

In our study, we morphometrically evaluated nine ovine cervical IVDs immediately after slaughter. To the best of our knowledge, this is the first study in recent literature to provide measurements of ovine cervical IVDs, and various dimensionswere obtained from the direct measurements in dissected discs. Epidemiologically, “Chiotikon” constitutes one of the dominant species in southern European countries, and is also one of the most productive ovine species worldwide. Our results indicated that the mean disc height featured an increase from cranial to caudal levels, which is generallyconsistent with the published data [22,24]. Anteroposterior and transverse diameters were almost equal in the majority of specimens, whereas a significant difference in thedisc thickness in the anterior surface in comparison with other surfaces was not observed. These data should be considered in the framework of demographic features, since the mean age and bodyweight of recruited specimens in our study was considerably less than the mean age of the Merino sheep utilized in previous investigations (Table 2).

The biomechanical evaluation of normal ovine cervical IVDs has also been the subject of specific investigations in the current literature [25,26,27,28,29,30]. The core characteristics of these investigations are summarized in Table 3.

Wilke et al. published the first documented data regarding the biomechanical profile of cervical intervertebral discs in the entire spine. It was reported that, in the flexion and extension of cervical spine, the greatest range of motion (ROM) was observed in the C1–C2 segment, featuring a notable numerical decrease in the underlying segment (C2–C3)with a subsequent progressive increase in caudal cervical segments (C3–C7). This pattern was also similar for the recorded ROM in axial rotation and lateral bending. Additionally, the results demonstrated that a neutral zone was the greatest in the cervical region instead of thoracic and lumbar, accounting for 50–70% for miscellaneous loading patterns [26].

In a subsequently published study, Long et al. [28] endeavored to investigate the multimodal effects of level, loading grade and injury on the biomechanical behavior of ovine cervical intervertebral discs. Studying forty-five cervical spine segments harvested from nine fresh-frozen ovine specimens, they found that the cranial levels (C2–C3 and C3–C4) were characterized by distinct torsional and axial responses when compared to caudal levels, principally by means of statistically significantly lesser axial ROM and tensile compliance, with no depicted difference regarding compressive compliance. These results were in general conformity with those published by Wilke et al. [26]. Furthermore, Long et al. reported that the torque range (defined as the peak-to-peak torque between the ±2° and ±4° angle of rotation) and torsional stiffness were considerably greater in the cranial than in the caudal levels, demonstrating a proportionate increase in response to the loading rate increment [28].

More recently, Derrouiche et al. attempted to shed more light on ovine cervical IVD complex biomechanics, studying the influence of osmotic conditions on its inelastic response. After the investigation of eighteen segments in a controlled environment, the potential chemo-mechanical coupling of recruited segments was assessed in compression, tension and torsion. The results demonstrated that chemo-mechanical interactions within an IVD microstructure are especially prominent in tension and compression loading, whereas torsional stiffness was observed to be chemically insensitive [29]. The impact of an osmotic environment in conjunction with other mechanical parameters such as pre-strain and compressive cyclic loading on ovine cervical IVDs viscoelastic properties was also verified from subsequently published reports [25,30].

In our study, a holistic biomechanical assessment of harvested specimens was performed. Considering the conclusions of similar experiments, we decided to shed more light on ovine cervical disc biomechanics by conducting—except for compressive cyclic testing—creep as well as compression to failure testing procedures. Regarding cyclic compression, it was observed that cycle loading is necessary for preconditioning in order for the force–displacement hysteresis response to be repeatable, a fact that has also been reported in previous investigations [25,29,30]. In their experiments, Derrouiche et al. [29] and Feki et al. [30] implemented ten cycles of compressive cyclic loading in order to accomplish preconditioning, a situation that is considered essential for the establishment of the chemo-mechanical equilibrium of the examined IVD. It was reported that, despite the fact that, in previous studies, several preconditioning compressive cycles were applied, the repeatability of mechanical response and, as a result, the reproducibility of experimental outcomes is present after cyclic compression testing in the first three cycles [30,32]. However, in our study, the consistent force–displacement correlation was only depicted to be accomplished at the end cycles of compression testing, which may be attributed to the different specimen preparation and biomechanical testing protocol. Regarding creep testing, our results were generally comparable to the respectively published studies for ovine lumbar discs, with the relatively lower total displacement of 0.08mm being primarily explained by the lesser applied load, which was 0.25 MPa in our study [33].

Finally, our experimental investigation was completed with the histological ex vivo evaluation. Generally, our findings regarding macro- and microscopic anatomy were found to be in accordance with the data from previous reports [31,34]. The typical structure of IVD, which consisted of the externally encountered fibrocartilaginous annulus fibrosus, the internally located cartilaginous nucleus pulposus and the superior and inferior cartilaginous endplates, was verified in our study. The staining of the specimens with Masson’s trichrome enabled the better identification of the biomechanically semantic complex concentric architecture of collagen lamellae in annulus fibrosus, whereas Alcian blue staining, interestingly, depicted the gradually decreasing concentration of proteoglycans from a central portion to the outer part of IVD, a phenomenon that is also observed in human IVD [1]. The presence of primitive notochordal cells in the nucleus pulposus represents a rather consistent finding considering existing reports. However, their morphological, biological, histochemical and immunohistological features as well as their overall role in ovine IVD degeneration warrants further investigation [34].

This particular study is characterized by specific methodological limitations, which may hinder the generalization of our results. First, a relatively limited number of specimens was utilized, not all of which were subjected to all evaluative tests. Second, the age, bodyweight and breed of selected animals should also be considered, since, despite the fact that the selected ovine breed is considered to be one of the most productive worldwide, established data regarding its life cycle and physical characteristics do not exist. Third, despite the dissection of assessed discs under magnification guidance and the verification of all specimen anatomic integrity prior to testing, microscopic bony remnants in the surface of dissected discs may contribute to the falsification of outcomes, especially regarding biomechanical investigation, a fact that was not highlighted in studies published to date.

In conclusion, this study aimed to multimodally determine the morphometric, biomechanical and histologic features of physiologic ovine cervical IVD as a unified structure, in the context of the paucity of such reports in current literature. To the best of our knowledge, this is the first investigation in the current literature evaluating directly dissected ovine cervical intervertebral discs and not the spinal segment as an integral unit. In general, our results were depicted to be in conformity with the literature findings, demonstrating, however, specific differentiations. Our data should be considered from researchers in the field of TE since selected animal models in these studies should be homologous, simulating possible human conditions [35]. Furthermore, the use of homologous large animal models provides more valid results regarding TE implants, minimizing confounders that may falsify results. Last but not least, the use of these models also facilitates the rational implementation of the 3Rs (reduction, refinement, replacement) principle, a standard that has not been strictly followed in current years [35,36].

## 4. Materials and Methods

### 4.1. Sample Preparation and Documentations

Three (*n* = 3) female sheep of the “Chiotikon” species (age range: 4–5 months, bodyweight range: 15–20 kg) were slaughtered for food production in a local abattoir. The cervical spine segments of the slaughtered animals were harvested for investigation, since these are theorized to be special by-products which are not allowed for human consumption according to the relative regulation (EC) No. 1774/2022 of the European Parliament and of the Council [31]. All aspects of this study were in accordance with the faculty ethics committee, and did not require official approval.

**Figure 7:**
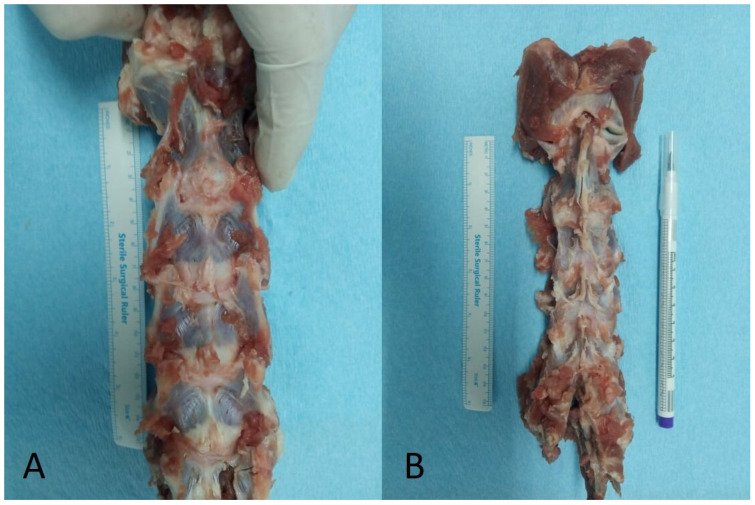
Anterior (**A**) and posterior (**B**) view of a harvested total cervical spine prior to the dissection of spinal segments.

Immediately after harvest, which was conducted directly after slaughter, total cervical spines were wrapped up at gauzes soaked with saline and stored in double plastic bags, which were frozen at −20 °C until experimentation [37]. Seven days after storage, they were thawed at room temperature and kept moist with normal saline. Subsequently, paravertebral musculature was carefully dissected so that the osseous and ligamentous structures were preserved (Figure 7). The C3–C4, C4–C5 and C5–C6 segments from each individual sample were selected to be included for final analysis due to their greater similarity to their human counterparts according to previous investigations as well as due to their ease of isolation [22]. Vertebrae were centrally cut and a total of nine (*n* = 9) spinal segments—including half of cranial vertebral body, half of caudal vertebral body and the intermediate IVD—were harvested for evaluation. After the meticulous removal of posterior osseoligamentous elements, IVDs were carefully dissected from the superior and inferior bony remnants of the adjacent vertebral body under the view of magnification loops (2.5×) using a high-speed drill (Midas REX, Medtronic^®^, Minneapolis, MN, USA) and a bone scraper. All harvested specimens were macroscopically anatomically intact, and considered eligible for further evaluation.

### 4.2. Morphometric and Biomechanical Evaluation

**Figure 8:**
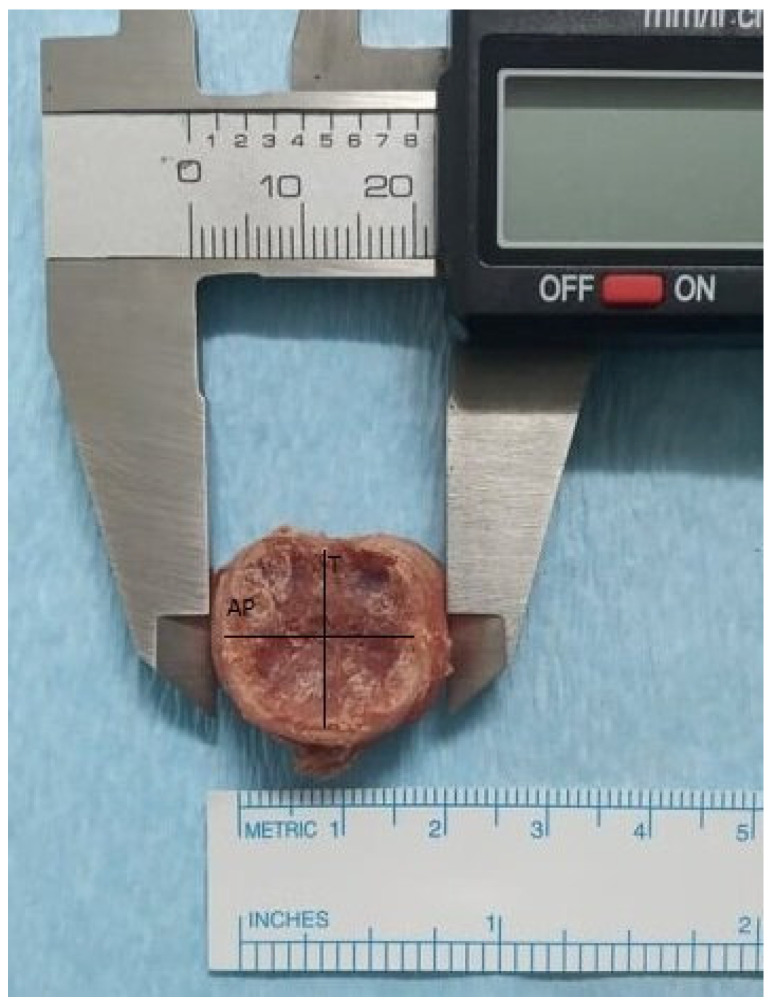
Morphometric evaluation of dissected intervertebral discs with digital ruler (AP: anteroposterior, T: transverse).

Dissected specimens were then subjected to morphometric evaluation using a digital ruler (measuring in millimeters and modified in order to represent an integer). Obtained measurements involved the anteroposterior (AP) diameter, the transverse (T) diameter and the maximal disc height in anterior (anterior disc height—ADH), posterior (posterior disc height—PDH), right (right disc height—RDH) and left (left disc height—LDH) surfaces (Figure 8).

After morphometric assessment, the specimens were transferred again at −20 °C for another week, and then subjected to another freeze–thaw cycle in isotonic saline (reference concentration of 9 g/L) at room temperature [25], before being transferred for biomechanical (*n* = 7) and histologic (*n* = 2) evaluation.

The biomechanical behavior of seven (*n* = 7) harvested specimens was investigated by performing uniaxial tests. The experiments were performed at room temperature on a universal testing machine (M500-50AT, Testometric, Lincoln, Lancashire, UK). The applied loads were registered through a 50 kN Newton loadcell (Testometric, Lincoln, Lancashire, UK) with a resolution of 0.01 N. The compressive force was converted to stress by dividing with the measured cross-section area of the disc, which is approximately 346 mm^2^. First, the selected specimens were subjected to twenty (20) cycle compression tests with a maximum load of 0.25 MPa, which corresponded to the pressure applied to the sheep when they were in a relaxed sitting state [37]. Cyclic loading was followed by a creep test where the disc was compressed under a constant load of 0.25 MPa for one hour. Finally, the compressive strength was measured by increasing the load with a constant rate of 3 mm/s and detecting the maximum stressreached on the obtained force–displacement curve.

### 4.3. Histological Evaluation

Two (*n* = 2) specimens were divided into five distinct slices, selecting the three intermediate slices for histologic analysis. Slices were routinely cut in 5 μm thick sections with a microtome, and subsequently formalin-fixed and paraffin-embedded followed by staining with (a) hematoxylin–eosin, (b) Masson’s trichrome and (c) Alcian blue, in concordance with the established histological procedures, performing one staining in each slice [34]. All slices were examined by a conventional light microscope (Zeiss^®^ Primotech Laboratory Microscope, Carl Zeiss Microscopy GmBH, Jena, Germany).

### 4.4. Statistical Analysis

Statistical analysis of retrieved data was conducted with statistical package Jamovi^®^ (Version 2.3, Computer Software). The recorded categorical variables (such as sex) were expressed as percentages, whereas continuous variables (as AP, T, ADH, PDH, RDH and LDH parameters) were expressed as the mean ± standard deviation using routine descriptive statistics. The normality of the collected data was examined with the Shapiro–Wilk test and the level of statistical significance was determined at *p* value = 0.05.

## Figures and Tables

**Table 1:** Representation of morphometric assessment data.

	Parameter	Overall Data Mean ± SD (95% CI)	Range	C3–C4 (Mean ± SD)	C4–C5 (Mean ± SD)	C5–C6 (Mean ± SD)	*p* Value (Shapiro–Wilk Test)
Measurement (mm)							
AP		24.4 ± 2.6 (22.4–26.4)	21.0–29.0	22.3 ± 1.5	24.3 ± 2.1	26.7 ± 2.5	0.808
T		24.4 ± 2.1 (22.8–26.1)	22.0–28.0	22.7 ± 0.6	24.0 ± 1.0	26.7 ± 2.3	**0.049**
ADH		7.1 ± 2.0 (5.6–8.7)	4.0–10.0	5.0 ± 1.0	7.3 ± 1.5	9.0 ± 1.0	0.779
PDH		6.6 ± 1.3 (5.5–7.6)	5.0–9.0	5.3 ± 0.6	6.3 ± 0.6	8.0 ± 1.0	0.407
RDH		7.7 ± 0.7 (7.1–8.2)	7.0–9.0	7.3 ± 0.6	7.7 ± 0.6	8.0 ± 1.0	**0.023**
LDH		7.4 ± 0.9 (6.8–8.1)	6.0–9.0	7.3 ± 0.6	7.0 ± 1.0	8.0 ± 1.0	0.337

SD: standard deviation; CI: confidence interval; AP: anteroposterior diameter; T: transverse diameter; ADH: anterior disc height; PDH: posterior disc height; RDH: right disc height; LDH: left disc height. Statistically significant *p* values are highlighted in bold. Level of statistical significance was determined at *p* value = 0.05.

**Table 2:** The principal characteristics of studies reporting the morphometry of normal ovine cervical intervertebral disc in the recent literature.

Authors	Year	Sample Characteristics	Breed (Age)	Comparative Sample	Measurement Methods	Conclusions
Cain and Fraser [23]	1995	Total cervical spine specimens (*n* = 7)	Merino (2 years)	(-)	Not specified	Ovine cervical spine anatomy is comparable to humans.
Wilke et al. [24]	1998	Total spine specimens (*n* = 5)	Merino (3–4 years)	(-)	Hand-held micrometer Goniometer	Ovine spine features significant similarities with those of humans regarding thoracic and lumbar spine anatomy, with specific limitations for cervical spine.
Kandziora et al. [22]	2001	Total cervical spine specimens (*n* = 20)	Merino (2 years)	Total human cervical spine specimens (*n* = 20)	Digital ruler Goniometer	Sheep cervical spine features favorable comparability with those of humans, constituting a valid model for cervical spine research.

**Table 3:** Core characteristics of studies investigating the biomechanical behavior of a normal ovine cervical IVD in recent literature.

Authors	Year	Sample Features	Breed (Age)	Outcome Measures	Evaluation Methods	Conclusions
Wilke et al. [26]	1997	Total spine specimens (*n* = 14)	Merino (4-year old)	ROM Neutral zone Stiffness quotient	Three-dimensional goniometric spine tester	Ovine spine features remarkable similarities with human, rendering it a satisfactory selection model for spinal implant assessment.
Gleizes et al. [27]	1998	Entire spine segment specimens (*n* = 19)	Not specified (2-year old)	Amplitude and rigidity in flexion/extension and lateral inclination	Experimental “2-TM” setup	Ovine spine segments freezing at −18 °C does not alter their biomechanical features, being a reliable method for specimen preservation prior analysis.
Long et al. [28]	2018	Cervical spine segment specimens (*n* = 45)	Swiss Alpine (2–5 years old)	Torque range Torsional stiffness Axial ROM Compliance	Servo-hydraulic material testing system (MiniBionix II 858, MTS Systems Corp., Eden Prairie, MN, USA)	Segment level, loading frequency and potential disc injury considerably influence ovine cervical IVD behavior.
Derrouiche et al. [29]	2019	Cervical spine segment specimens (*n* = 18)	Not specified (8–10 months)	Load–displacement curves (tension, compression and torsion) under various NaCl concentrations	Displacement-controlled universal axial testing machine (Instron-5500) Angle-controlled universal torsion testing machine (Instron-8874)	Torsional stiffness was observed to be chemically independent in contrast to compression and tension, advocating for the chemo-mechanical coupling of ovine cervical IVD.
Derrouiche et al. [25]	2020	Cervical spine segment specimens (*n* = 15)	Not specified (12 ± 1 months)	Axial and torsional responses with or without pre-strain under various NaCl concetrations	Universal testing machine (Instron-8874)	Pre-strain significantly affects the interactions between torsional response and the internal osmotic pressure of ovine cervical IVD.
Feki et al. [30]	2020	Cervical spine segment specimens (*n* = 8)	Not specified (1–2 years)	Effect of a normal axial cyclic compression on viscoelastic properties of cervical IVD	Universal axial testing machine (Instron-5500)	Compressive cyclic loading and surrounding osmotic conditions remarkably affect the viscoelastic behavior of ovine cervical IVD.

IVD: Intervertebral disc; ROM: Range of motion; NaCl: Sodium chloride.

## Data Availability

The original contributions presented in this study are included in the article, and further inquiries can be directed to the corresponding author/s.
